# Disentangling linkages between satellite-derived indicators of forest structure and productivity for ecosystem monitoring

**DOI:** 10.1038/s41598-024-64615-2

**Published:** 2024-06-14

**Authors:** Evan R. Muise, Margaret E. Andrew, Nicholas C. Coops, Txomin Hermosilla, A. Cole Burton, Stephen S. Ban

**Affiliations:** 1 Department of Forest Resource Management, University of British Columbia, 2424 Main Mall, Vancouver, BC V6T 1Z4 Canada; 2 Centre for Terrestrial Ecosystem Science and Sustainability, Murdoch University, 90 South St, Murdoch, WA 6150 Australia; 3 Canadian Forest Service (Pacific Forestry Centre), Natural Resources Canada, 506 West Burnside Road, Victoria, BC V8Z 1M5 Canada; 4 BC Parks, Ministry of Environment and Climate Change Strategy, Stn Prov Govt, PO Box 9360, Victoria, BC V8V 9M2 Canada

**Keywords:** Macroecology, Forest ecology, Ecosystem ecology

## Abstract

The essential biodiversity variables (EBV) framework has been proposed as a monitoring system of standardized, comparable variables that represents a minimum set of biological information to monitor biodiversity change at large spatial extents. Six classes of EBVs (genetic composition, species populations, species traits, community composition, ecosystem structure and ecosystem function) are defined, a number of which are ideally suited to observation and monitoring by remote sensing systems. We used moderate-resolution remotely sensed indicators representing two ecosystem-level EBV classes (ecosystem structure and function) to assess their complementarity and redundancy across a range of ecosystems encompassing significant environmental gradients. Redundancy analyses found that remote sensing indicators of forest structure were not strongly related to indicators of ecosystem productivity (represented by the Dynamic Habitat Indices; DHIs), with the structural information only explaining 15.7% of the variation in the DHIs. Complex metrics of forest structure, such as aboveground biomass, did not contribute additional information over simpler height-based attributes that can be directly estimated with light detection and ranging (LIDAR) observations. With respect to ecosystem conditions, we found that forest types and ecosystems dominated by coniferous trees had less redundancy between the remote sensing indicators when compared to broadleaf or mixed forest types. Likewise, higher productivity environments exhibited the least redundancy between indicators, in contrast to more environmentally stressed regions. We suggest that biodiversity researchers continue to exploit multiple dimensions of remote sensing data given the complementary information they provide on structure and function focused EBVs, which makes them jointly suitable for monitoring forest ecosystems.

## Introduction

Monitoring the changing state of biodiversity globally is key to understanding and mitigating the increased extinction risk for many species^1,2^ and the homogenization of biotic communities at various spatial and temporal scales^3^. The Group on Earth Observations Biodiversity Observation Network has developed the Essential Biodiversity Variables (EBVs)^4^, which are standardized, comparable variables that represent a minimum set of biological information required for monitoring biodiversity change at large spatial extents^5^. The EBVs are designed as an analog to the Essential Climate Variables framework^6^ to be global in scope, relevant to biodiversity information, feasible to implement and use, and complementary to one another^5^. There are six classes of EBVs collected at various scales. The ecosystem EBV classes (ecosystem structure and ecosystem function) are well suited to be monitored across large areas using remote sensing technologies^5^. This has led to a proliferation of potential EBV datasets that have been generated to describe these two ecosystem EBV classes, especially in forested environments.

Ecosystem structure is the composition, abundance, and spatial arrangement of different ecosystem components, and is indicative of habitat quality and connectivity^7^. Forest ecosystem structure is defined as the geographic distribution of trees or biomass, with forest structural diversity encompassing the measurable morphological components of the forests (e.g., canopy cover, canopy height, and structural complexity)^8,9^. Forest structural diversity has been linked to species richness at various spatial scales, ranging from individual plots to landscapes^10,11^. A variety of forest structural attributes can be extracted directly from lidar (light detection and ranging) data, ranging in complexity from simple (e.g., canopy cover, canopy height) to more complex (e.g., foliage height diversity, leaf area index), while others can be indirectly modelled from lidar and auxiliary data (e.g., aboveground biomass, basal area)^12^. A suite of these, and other, structural attributes have been used as indicators of biodiversity at local to landscape scales^10,13–15^. Increased forest structural complexity has been hypothesized to create additional niches, leading to increased species diversity^10,16^, which has been frequently demonstrated using avian species diversity metrics^17^ and lidar^18^, or a combination of lidar and satellite imagery^19^, with some research indicating that canopy vertical distribution was the strongest predictor of species richness^20^. Although lidar datasets have traditionally been local in extent, advances in satellite remote sensing processing have allowed the extension of three-dimensional forest structure attributes nationally in a wall-to-wall fashion. The computation of these attributes often requires data fusion approaches involving lidar data and medium spatial resolution optical imagery^12,21,22^.

Ecosystem functions are measures of ecosystem performance that are the consequence of one or multiple ecosystem processes^23,24^. Within the EBV framework, vegetation phenology—the timing and duration of leaf-on conditions—and ecologically available energy—the amount of accessible and useable energy available for growth in an ecosystem—are useful predictors of species richness and abundances at various scales^25–28^. Ecologically available energy is the amount of energy produced in an ecosystem, which is sensitive to limitations such as recent disturbances and the water and nutrient supplies, and is measurable via productivity^29,30^. This differs from ambient energy in that it goes beyond the climatic conditions of an area, and concerns the amount of resources available for consumption in an ecosystem^31^. Clear links have been developed between remote sensing-derived spectral reflectance, particularly in the near-infrared and the red parts of the electromagnetic spectrum^32^, which relate to pigment concentrations and leaf structure, respectively, as well as productivity^33^. Consequently, the normalized difference vegetation index (NDVI) and other vegetation indices have been correlated with productivity measures^34,35^. The relationship however, is typically non-linear, principally due to saturation in the spectral response at moderate to high leaf area index values^36^. As a result, the strength of the relationship between spectral indices and productivity depends on the ecosystem type, land cover type, amount of canopy cover, and productivity in the region^37^. Likewise, relationships between productivity indicators and biodiversity also contain uncertainty, recognizing that biodiversity is governed by a variety of factors of which productivity is but one^5^. Despite these uncertainties, remotely sensed vegetation indices have enabled the assessment of biodiversity patterns at single time points^29,38,39^, or through time when using time series data^26,27,40^.

While biodiversity is not solely driven by productivity, the relationship between productivity and biodiversity can be explained through various hypothesized mechanisms: including available energy, environmental stress, and environmental stability. These three hypotheses have been used to guide the development of a set of remote sensing indicators, the Dynamic Habitat Indices (DHIs), which summarize intra-annual dynamics in productivity metrics and have been evaluated as broad-scale biodiversity indicators globally for a variety of taxa^26,40,41^. According to the available energy hypothesis^29,42^ an increase in ecologically available energy results in higher species richness. In the context of the DHIs, the total energy available within a specific area is quantified using the cumulative DHI, which sums each productivity observation throughout the course of a year. The environmental stress hypothesis^43^ suggests that higher levels of minimum available energy contribute to greater species richness. The minimum DHI, derived from the lowest productivity observed over the course of a year, indicates the level of available energy during stressful periods. The environmental stability hypothesis^44^ proposes that lower energy variation throughout the year leads to increased species richness due to reduced resource bottlenecks. The variation DHI captures the coefficient of variation in a vegetation index over the course of a year, reflecting the prevalence of resource bottlenecks. The DHIs have previously been produced at continental to global extents using Moderate Resolution Imaging Spectroradiometer (MODIS) imagery, while recent research has focused on producing the DHIs at finer spatial resolutions using multi-annual time series of Landsat imagery^45^.

The DHIs have been shown to be correlated with aspects of biodiversity across a variety of spatial scales and locations, including moose occurrence and abundance in Ontario, Canada^46^ and across Russia^47^, beta diversity in butterflies in Canada^48^, avian diversity in the coterminous U.S.^49^, Ontario^50^ and France^51^, and global alpha diversity^26^. The DHIs have also been used to construct novel ecoregionalizations^52,53^. DHIs, however, are not able to capture all components of biodiversity due to the many environmental and landscape processes not observable from satellite data^50^.

The suite of remote sensing indicators used in biodiversity monitoring should be carefully considered. Ideally, indicators should be selected that are closely related to the phenomenon of interest to allow for direct integration of monitoring results with management actions^54^. With the advent of large-extent monitoring methods like satellite remote sensing, which have led to a proliferation of potential EBV datasets, it becomes important to assess the interrelationships between these datasets and the complementarity of the information they provide to reduce redundancy in future EBV development efforts^4,5^. Identifying and exploring the strong relationships that may exist between indicators of EBVs may help elucidate the ecological relationships between these facets of biodiversity, such as ecosystem structure and function, at local to global scales. On the other hand, when remote sensing-derived EBVs are not related, they may be well suited to be used in monitoring programs together, as complementary sets of observations.

The relationships between ecosystem structure and function have been examined within a remote sensing context for decades^36,55,56^. Hypothesized mechanisms such as niche complementarity suggest that productivity increases with stand structural complexity^57^, while asymmetric competition for light can reduce forest productivity with increased structural complexity^58^. Some aspects of forest structure, such as canopy cover and leaf area index, also directly influence vegetation indices, which are used to estimate productivity^59^. Often, these large-extent estimates of structure and function metrics are generated from similar data sources, such as the Landsat series of satellites^21,22,26,45^. Due to this shared data provenance, and the linkages between biodiversity and both structure and function, it is important to assess how much unique information these structure and function metrics represent when used as indicators of biodiversity.

The goal of this study is to assess the complementarity of two remote sensing datasets often proposed as indicators of ecosystem structure and function EBVs. We interpret weak or absent relationships between metrics to constitute complementary information. To do so, we synthesize wall-to-wall forest structure data previously generated by modelling lidar-derived forest structure metrics with medium spatial resolution satellite imagery using a k-NN imputation method^21,22^, alongside a well-established remote sensing-derived index of ecosystem function (DHIs)^26,40,41,45^. First, we analyze remote sensing indicator complementarity by examining the multivariate relationship between ecosystem structure and function. Second, we propose a method for selecting which remote sensing attributes to develop and apply within a single EBV class by determining the independent and shared contributions of lidar-derived forest structural attributes and modelled forest structure to explaining ecosystem function. Third, we examine the potential geographic extensibility of these results by evaluating the consistency of the observed forest structure–function relationships across a broad range of environment gradients and forest types.

## Materials and methods

### Study area

British Columbia (BC) is the westernmost province of Canada, and is home to a variety of terrestrial ecosystems. Located between the Pacific Ocean and the Rocky Mountains, approximately 64% of the province is forested, with large environmental and topographic gradients^60,61^. The majority of the forests of BC are coniferous forests (87.4%), with the remaining forests being broadleaf (8.9%), wetland-treed (2.7%) and mixed-wood (0.9%)^62^. There is a large topographic gradient in the province, with elevations ranging from sea level to over 4000 m, with much of the province being mountainous (elevations above 1000 m)^63^. Wildfires are a regular disturbance in the province, although larger, higher intensity fires have become more common in recent years due to disruption of the historical fire regime^64^. In addition, forestry is a large industry in the province, with approximately 136,000 ha of the province harvested accounting for ~ 54 million cubic metres of volume of wood in 2021^65^.

**Figure 1:**
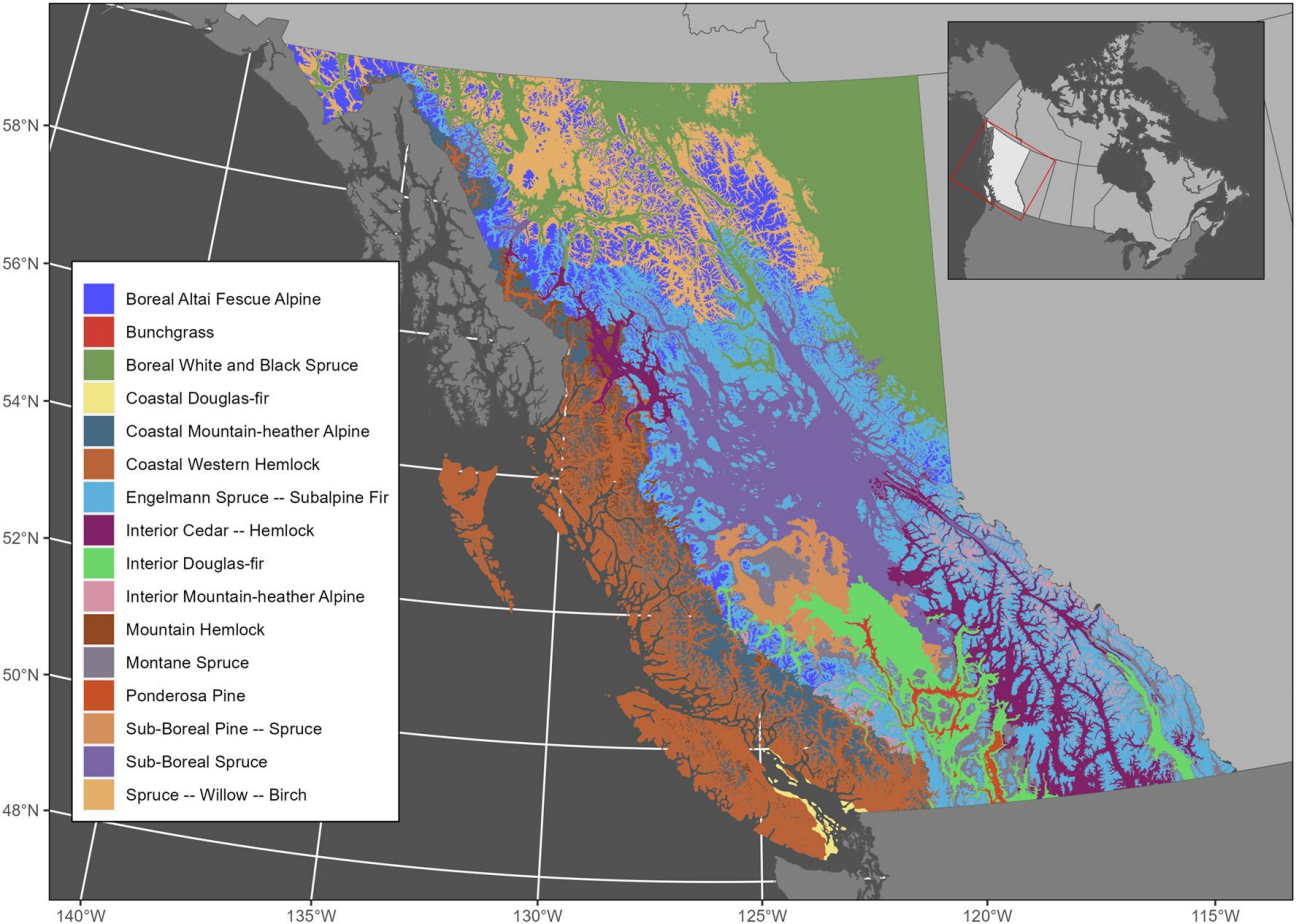
Location of biogeoclimatic ecosystem classification (BEC) zones in British Columbia. Map was generated in R version 4.3.1 using the ggplot2 version 2.1.0^99^ and sf version 1.0–14^100^ packages.

BC is stratified into 16 zones based on the dominant tree species and climate by the Biogeoclimatic Ecosystem Classification (BEC) system (Fig. 1). To examine trends across the large environmental gradients, we group the BEC zones into five broad biomes: the Northern interior, Southern interior, Montane, Alpine, and Coastal groups, similar to Hamann et al.^66^. We also report the average climate data for BEC zones from 1991 to 2020, according to Wang et al.^67^ (Table 1).

**Table 1:** Biogeoclimatic ecosystem classification (BEC) Zones, their aggregated biomes, monthly average climate values for precipitation, maximum temperature, and minimum temperature, and the number of samples taken from each BEC zone.

BEC group	BEC zone	Full name	Average monthly precipitation (mm/year)	Average monthly max temperature (°C)	Average monthly min temperature (°C)	n
Alpine	BAFA	Boreal Altai Fescue Alpine	1357.3	1.9	− 5.6	500
Alpine	CMA	Coastal Mountain-heather Alpine	2878.1	4.5	− 2.4	500
Alpine	IMA	Interior Mountain-heather Alpine	1621.7	3.1	− 4.2	173
Coastal	CDF	Coastal Douglas-fir	986.1	12.9	5.9	500
Coastal	CWH	Coastal Western Hemlock	2518.8	10.6	3.5	500
Montane	ESSF	Engelmann Spruce–Subalpine Fir	1087.6	5.3	− 3.1	500
Montane	MH	Mountain Hemlock	2666.2	7.2	− 0.2	500
Montane	MS	Montane Spruce	622.1	8.3	− 2.5	500
North	BWBS	Boreal White and Black Spruce	516.3	5.9	− 5.3	500
North	SBPS	Sub-Boreal Pine–Spruce	480.1	9.0	− 3.7	500
North	SBS	Sub-Boreal Spruce	619.1	8.1	− 2.3	500
North	SWB	Spruce–Willow–Birch	730.7	3.6	− 5.7	500
South	BG	Bunchgrass	320.0	13.1	1.4	168
South	ICH	Interior Cedar–Hemlock	901.4	9.5	− 0.3	500
South	IDF	Interior Douglas-fir	476.8	10.6	− 0.7	500
South	PP	Ponderosa Pine	352.2	13.6	2.9	478

Climate data from Wang et al. (2016) averaged for 1991–2020.

### Data

#### Forest structure

The forest structural attributes (canopy height, canopy cover, structural complexity [coefficient of variation of height returns], basal area, aboveground biomass, and gross stem volume) were retrieved for 2015 from the 30 m spatial resolution dataset generated by Matasci et al.^21,22^. Their method extends structural attributes derived from a set of lidar acquisitions and field plots across Canada. These data were generated for the treed pixels of Canada using the BAP Landsat surface reflectance composite^68^ and auxiliary data (i.e., topography, geography) using a k-Nearest Neighbour imputation^21,22^. Accuracy metrics for imputed forest structural attributes range from an RMSE of 29.7% (structural complexity) to 82.3% (gross stem volume) and *R*^2^ ranging from 0.125 (structural complexity) to 0.712 (gross stem volume)^21,22^.

#### Dynamic habitat indices

The DHIs for the terrestrial area of BC were calculated following the procedures described in Razenkova et al.^45^. Although DHIs are typically derived from high-temporal, coarse spatial resolution imagery such as MODIS, we used Landsat surface reflectance data due to its finer spatial resolution, which corresponds to the 30 m ALS derived forest structure data. To account for the lower temporal resolution of Landsat, we generated a synthetic year of monthly data from a ten-year timespan of Landsat imagery. Google Earth Engine (GEE)^69^ was used to obtain all valid Landsat pixels for the study area over a ten-year period centered on 2015. Selected pixels were filtered for shadows, clouds, and cloud shadows using the QA band derived from the fmask algorithm^70^, available on GEE. The NDVI was then calculated for each pixel in each image and summarized into a synthetic year of monthly data by calculating the median of each month’s NDVI values, disregarding the year of the image. The calculations for the Cumulative DHI, Minimum DHI, and Variation DHI can be found in Table 2, alongside a summary of the main datasets analyzed in this study.

**Table 2:** Datasets used in this study.

Dataset	Metric group	Name	Date	Metric	Sources
Forest structure	Primary; extracted directly from the lidar data	Canopy height	2015	95th height percentile of lidar returns	Matasci et al. ^21,22^
		Canopy cover	2015	Proportion of lidar returns above 2m	
		Structural complexity	2015	Coefficient of variation of lidar returns	
	Modelled; Modelled based on the lidar data and field information	Gross stem volume	2015	Total volume of trees/lidar plot	
		Basal area	2015	Total cross sectional tree area/lidar plot	
		Aboveground biomass	2015	Total tree biomass/lidar plot	
Dynamic habitat index (DHI)	Response variables	Cumulative DHI	2011–2020	Sum of synthetic year of monthly NDVI observations $\text{Cumulative DHI}=\sum_{i=1}^{12}{\text{x}}_{i}$	Radeloff et al.^26^ Razenkova et al. ^45^
		Minimum DHI	2011–2020	Minimum of synthetic year of monthly NDVI observations $Minimum DHI = min\{{x}_{1},{x}_{2}, ..., {x}_{12}\}$	
		Variation DHI	2011–2020	Coefficient of variation of synthetic year of monthly NDVI observations $\text{Variation DHI}=\frac{\sigma \left(x\right)}{\overline{x}}$	

All datasets derived at 30 m spatial resolution. Forest structural attributes were generated from lidar returns at the plot level then imputed across the study area using Landsat data. Equations for the DHIs have x denote the median of monthly NDVI observations using the synthetic year of data.

#### Ancillary data

##### Surface-reflectance image composites

The forest structure, forest disturbance, and land cover layers are based on a 30 m best-available-pixel (BAP) composite generated by Hermosilla et al.^68^ using the Composite2Change approach. The composites were developed for each year from 1984 to 2019 by selecting the best available imagery from the available growing season Landsat observations, removing pixels with clouds, cloud shadows, and haze. The methodology uses the scoring method from White et al.^71^ to select the best pixel for a given year from Landsat-5 Thematic Mapper, Landsat-7 Enhanced Thematic Mapper Plus, and Landsat-8 Operational Land Imager imagery. A spectral trend analysis was conducted over these initial BAP composites using the Normalized Burn Ratio (a vegetation index) on each pixel to remove unscreened noise, detect changes, and temporally interpolate data gaps. This resulted in a gap-free surface-reflectance composite across Canada^72^. The forest structure and land cover datasets are based on BAP data from 2015, while the forest disturbance mask covers the period from 1985 to 2020.

##### Forest disturbances

We used the forest disturbance layer generated by Hermosilla et al.^68^ to restrict the analysis to stands older than 35 years as productivity and structure metrics have been shown to be strongly decoupled in early regenerative stands^73^, and to exclude recently anthropogenically disturbed stands. The disturbances detected using spectral trend analysis were attributed to a disturbance agent (i.e., wildfire, harvest) within an object-based analysis approach using a Random Forests classification model^74^. Changes were detected with an overall accuracy of 89%, attributed with an accuracy of 92%, and assigned to the correct year with an accuracy of 89%^68^.

##### Land cover

We used the land cover map for 2015 generated by Hermosilla et al.^62^ to stratify our samples alongside the BEC zones. The 30 m spatial resolution land cover map for Canada’s forested ecosystems was generated using the Virtual Land Cover Engine framework^75^. This framework uses a Random Forests based classification approach, and integrates logical land cover transitions using a Hidden Markov Model to generate 12 land cover classes. The land cover classification reached an overall accuracy of 77.9% ± 4%^62^. We restricted our sample to the four treed land cover classes: broadleaf, coniferous, mixed wood, and wetland-treed.

### Sampling

To obtain representative sample units of the EBV metrics across the study area, we implemented stratified random sampling across the union of BEC zones (Table 1) and forest types (i.e., coniferous, broadleaf, mixed wood, and wetland treed; see “Study area” section) of BC. Sample units were restricted to forested pixels that were surrounded by the same forest type to reduce the chance of sampling mixed pixels. Sample units were selected from homogeneous areas to reduce uncertainty associated with the input datasets^76^. Homogeneous areas were defined as 3 × 3-pixel windows with a coefficient of variation for canopy cover and canopy height below 0.5. A minimum sampling distance of 1 km was implemented to reduce the effects of spatial autocorrelation. Sample units that were disturbed in the last 35 years were excluded by using the disturbance mask generated for the forested ecosystems of Canada^68^. A maximum of 500 sample units were selected from each stratum. In the strata where this was not achieved, all sampling units meeting the above restrictions were selected (see Table 1). The sampling was conducted in R version 4.2.2^77^ using the sgsR package^78^. Neighbourhood analyses for the land cover classes and coefficient of variations of canopy height and cover were calculated in Python version 3.9.

### Analysis

Redundancy analysis (RDA) and variation partitioning were used to relate the primary and modelled forest structure variables to ecosystem function across a broad environmental range. RDA has widely been used in community ecology where environmental variables of interest are compared to species composition^79,80^. RDA predicts multiple response variables by first running a multiple linear regression of the predictor variables on each response variable, and then a principal component analysis on the fitted values from each multiple linear regression. This reduces the dimensionality of the fitted values by transforming them into a set of independent RDA axes, and allows the strength of the multivariate relationship between predictors and responses to be assessed by calculating eigenvalues of the RDA. Relationships between variables are revealed by the loadings of both predictor and response variables on the RDA axes. RDA axes are labelled as RDA1, RDA2, etc. to indicate they are derived from redundancy analysis, rather than other ordination techniques such as principal component analysis or constrained correspondence analysis^81^. Partial RDA extends RDA by allowing groups of predictor variables to be considered as co-variates. Variation partitioning leverages multiple partial RDAs to assess the overlap between the explanatory power of the groups of predictor variables^81^.

We performed RDAs for our full sample, pooling BEC zones and forest types, as well as individually by BEC zone and forest type, with results aggregated to BEC zone groups (Table 1). All measured values were natural-log transformed and standardized to Z-scores based on the summary statistics of the full sample prior to analyses. Following the data transformation, we visually assessed the pairwise relationships between the overall and stratified datasets for the assumptions of linearity and homoskedasticity. Forest structure variables were treated as predictor variables and were grouped based on whether they were primary estimates from the lidar data or modelled from lidar and ancillary information (Table 2) for variance partitioning. The three DHIs were used as the response variables. Analysis of variance tests were used to determine which RDA axes were significant, with p-values below 0.1, using an F-test. The proportion of variance attributable to each axis was calculated from the eigenvalues generated by the RDA, and axis loadings were calculated as the Pearson correlation between each forest structure or DHI variable and the RDA axes. To ensure similar directionality (+ or −) in the loadings across each variable in every strata, we transformed the data such that the cumulative DHI loading was always positive. To do so, if the cumulative DHI loading was negative we multiplied each loading in that strata and axis by − 1. Forest structural attribute axis loadings represent the strength of relationship between a given variable and the RDA axis, while the DHI loadings indicate what is being represented by the RDA axes. To visualize the RDA results, we plot the significant loadings as annotated arrows between the predictor/response variables and each RDA axis (shown as a box), similar to a path diagram. We also display the amount of variance explained by each RDA axis within their respective box.

Variation partitioning is displayed using a Venn diagram in which the percentage of variance explained by each dataset is in a circle, and the overlap between circles represents the overlap in variance explained. We add paths to the Venn diagram to indicate how the structural attributes contribute the variation in the DHIs. Because the variation partitioning results summarize the full multivariate solution, rather than individual RDA axes, we present variation partitioning results alongside the path diagrams illustrating the details of the RDA results. RDA and variation partitioning calculations were done in R^77^ version 4.2.2 using the vegan package^82^. The code associated with the processing and analysis is available at https://github.com/emuise/code-structProdSem.

**Figure 2:**
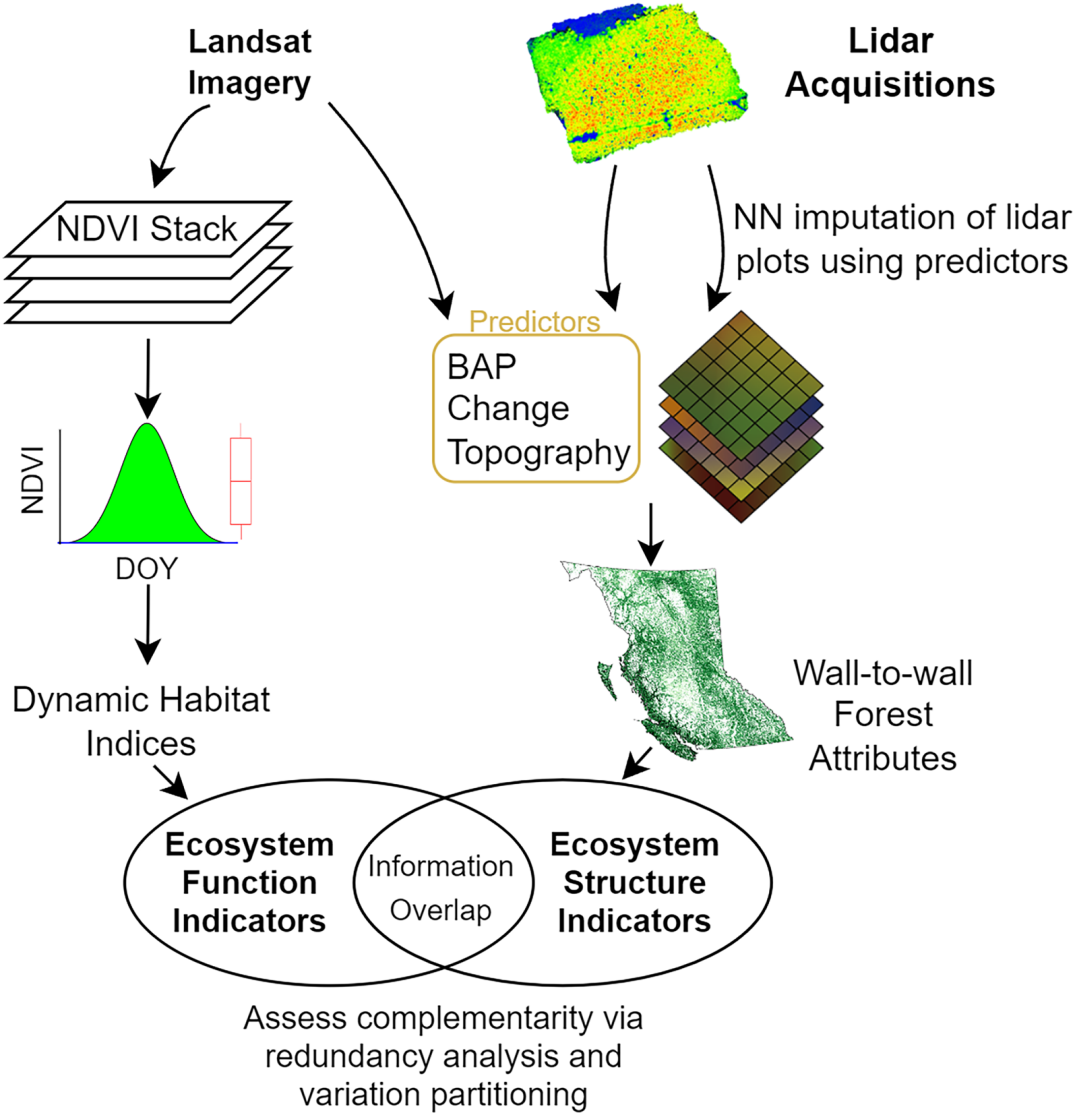
Flow diagram of the data to indicators to results pipeline implemented in this study.

We present a simplified flow diagram of the data provenance to result pipeline in Fig. 2.

## Results

**Figure 3:**
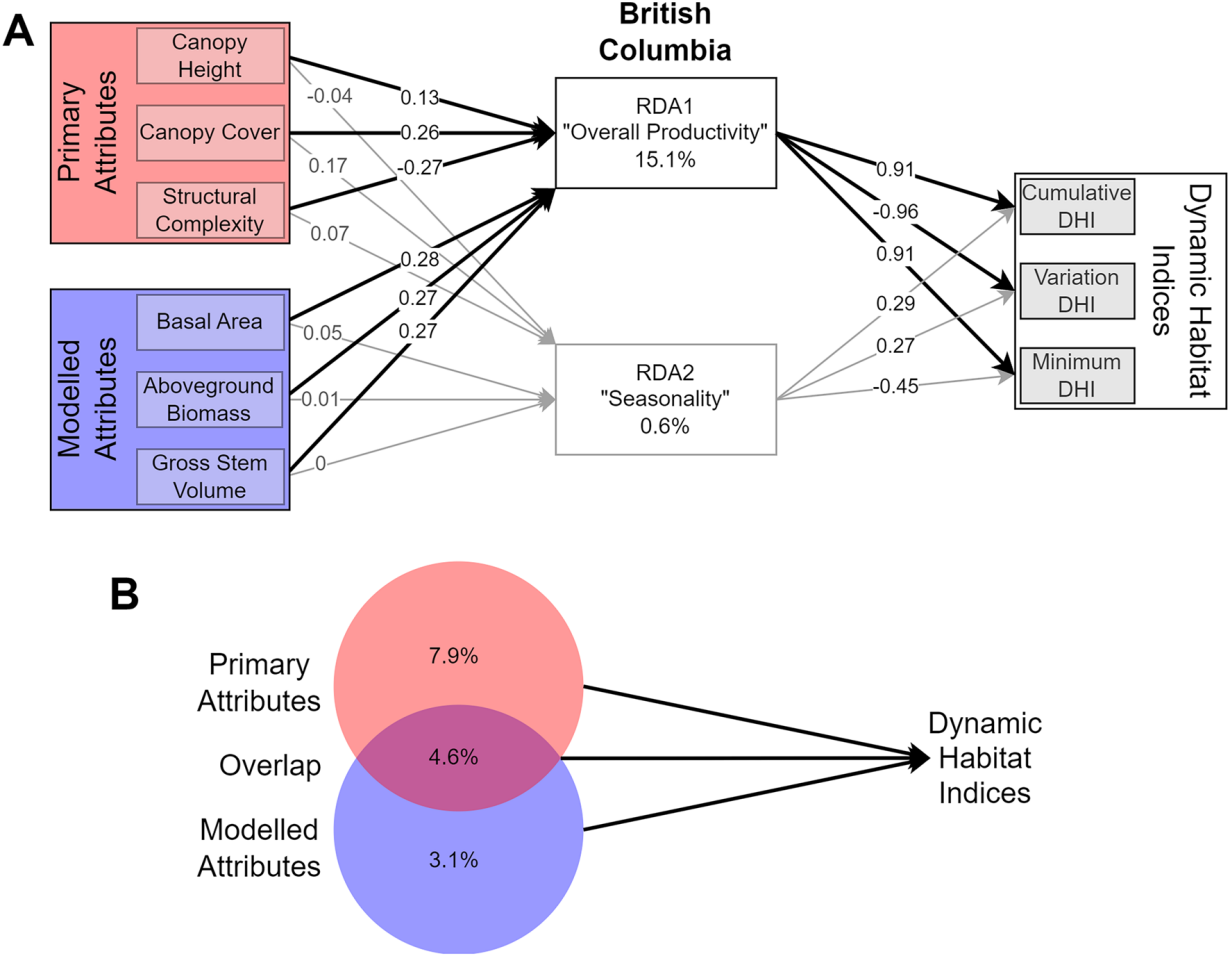
(**A**) Axis loadings from redundancy analysis (RDA) of primary and modelled forest structure variables on the dynamic habitat indices (DHIs). (**B**) Results from variation partitioning of primary and modelled forest structure variables on the DHIs. Both visualized analyses are across the entire dataset. See Supplementary Information for results from each Biogeoclimatic Ecosystem Classification zone and forest type.

Overall in BC, the intra-annual patterns of productivity as represented by the DHI variables were weakly related to forest structure (Fig. 3). Less than 16% of the overall variation in the DHIs was explained by the forest structural attributes. Due to strong correlations between the axes of the DHIs, the majority of the variation was held within the first RDA axis, which had strong loadings for all DHIs, with positive loadings for the cumulative and minimum DHIs, and negative loadings for the variation DHI. As such, we identified the RDA1 axis as representing overall productivity. The overall productivity axis had similar loadings (between 0.26 and 0.28) for all modelled forest structural attributes and canopy cover. Canopy height had a smaller loading (0.13), while structural complexity was negatively correlated with the overall productivity axis. RDA2 explained much less of the variation in the DHIs. RDA2 had a large negative loading on the minimum DHI (− 0.47), with smaller, positive loadings on the cumulative (0.26) and variation (0.29) DHIs. Due to the minimum DHI having the largest influence on RDA2, we identified it as a seasonality axis. Canopy cover and structural complexity had the strongest (0.18 and 0.08, respectively) loadings on the seasonality axis (Fig. 3A). Variation partitioning highlighted that the majority of the explained variation in the DHIs was due to the primary forest structural attributes (canopy cover, canopy height, and structural complexity; 7.7% variation explained). The overlap between primary and modelled attributes was 4.2%, and the modelled attributes explained 3.4% of the variation on their own (Fig. 3B).

**Figure 4:**
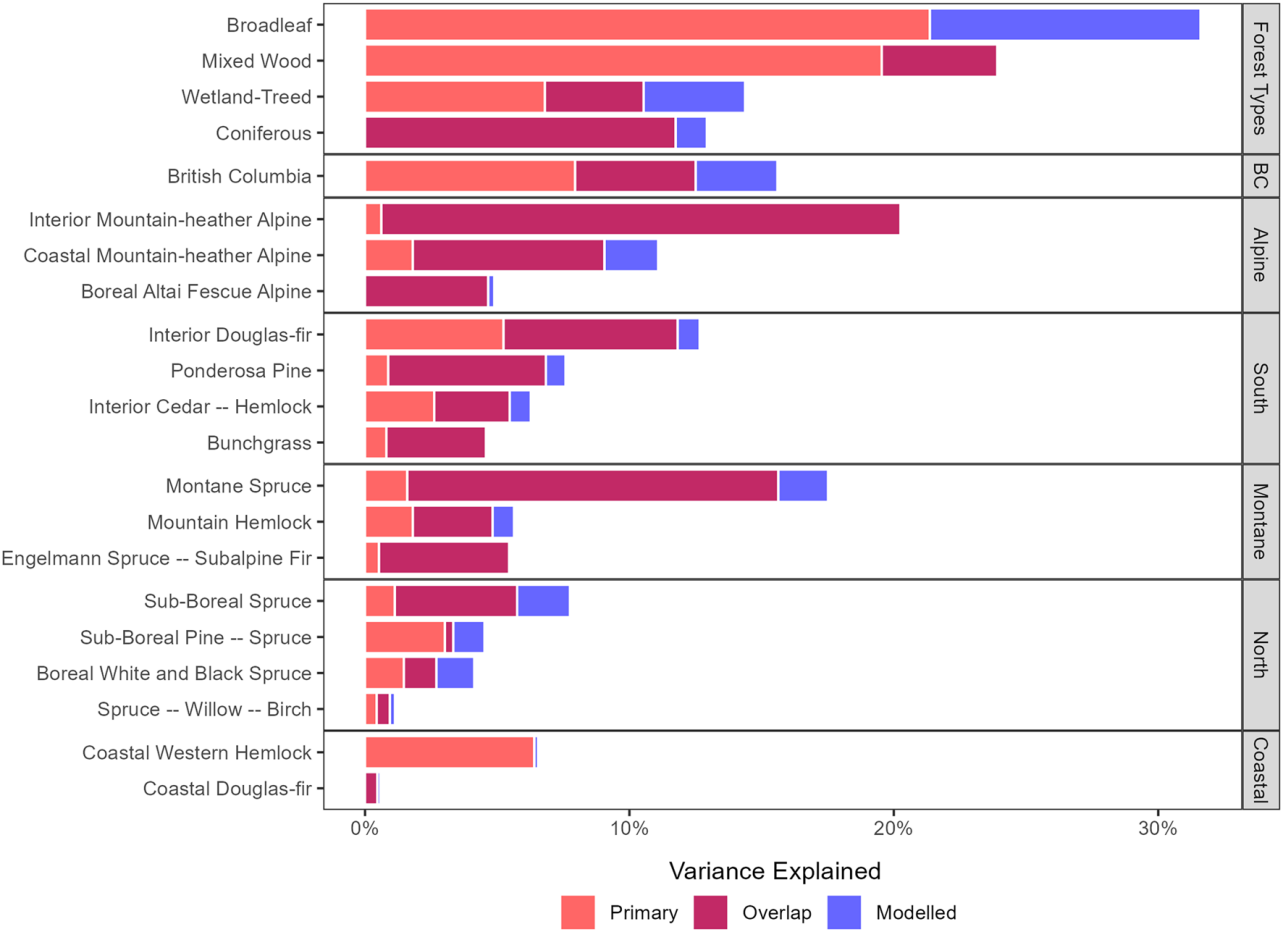
Stacked bar plot showing proportion of the variation in the dynamic habitat indices (DHIs) explained by primary, modelled, and the overlap between primary and modelled structure variables across the stratified and overall datasets.

By forest type, results indicated similar or increased amounts of variance explained when compared to the overall dataset (Fig. 4). By BEC zone, results generally indicated smaller amounts of variation explained, which may be due to the relatively small environmental gradients within the zones. The overlap between the primary and modelled datasets was often responsible for the majority of the variation explained in the DHIs, except when stratifying by forest types, where this was only true for coniferous forests. For broadleaf, mixed wood, and wetland-treed forest types most of the explained variation in the DHIs was contributed by the primary forest structural attributes. A similar pattern was found by BEC zones, where the variation in the DHIs was principally explained by the overlap between primary and modelled forest structural attributes, except for CWH, where the variation was entirely explained by the primary forest structural attributes. The Coastal BEC group had the lowest amount of variation explained, with Coastal Douglas-fir having < 1% of variation explained by the structural data. Overall, the DHIs were decoupled from the forest structural attributes, as shown by the overall variation explained being under 32% regardless of data stratification.

**Figure 5:**
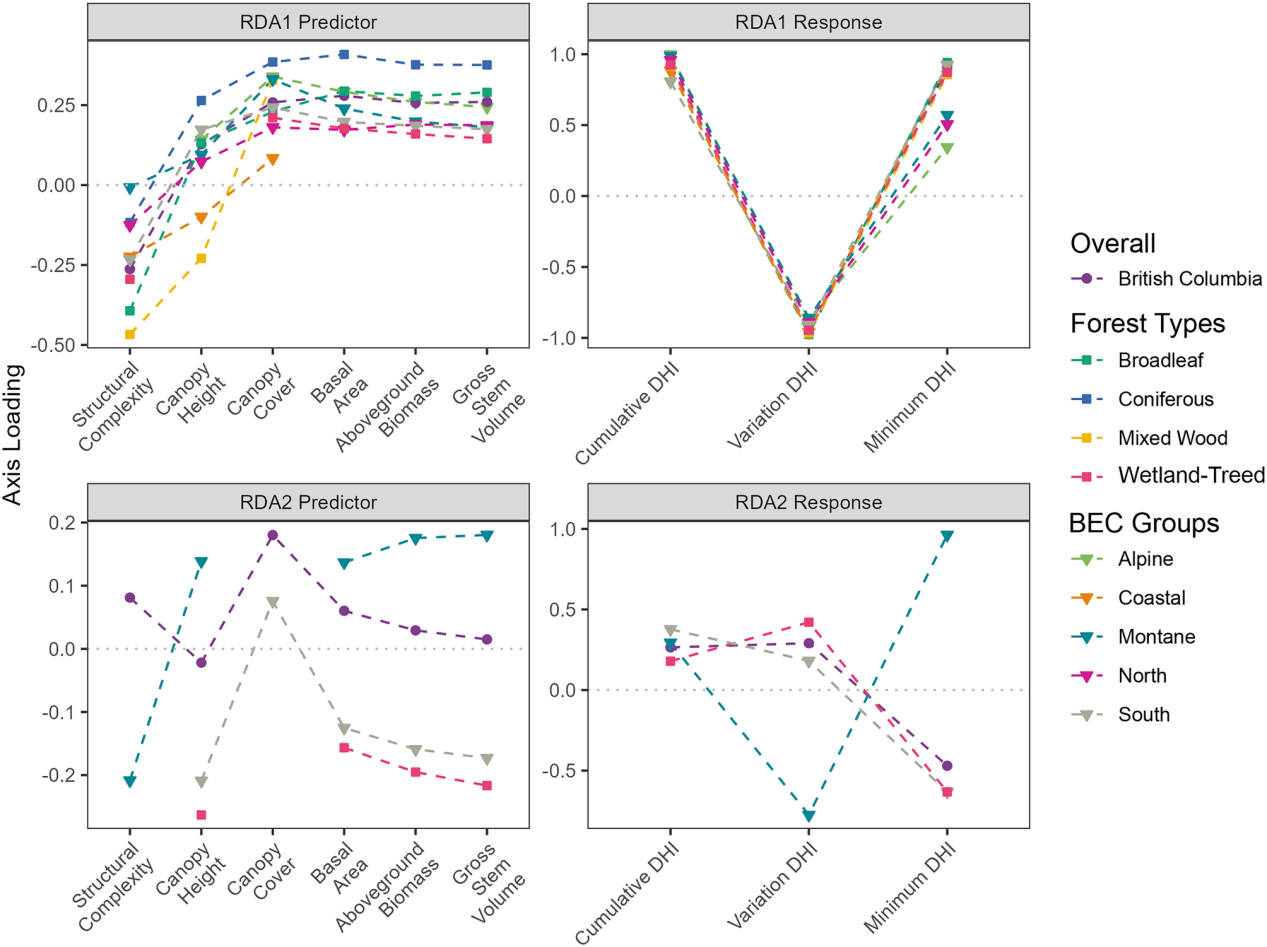
Parallel coordinate plots of average significant loading strength by Biogeoclimatic Ecosystem Classification (BEC) group, forest type, and British Columbia. Note the varying y-axes.

**Figure 6:**
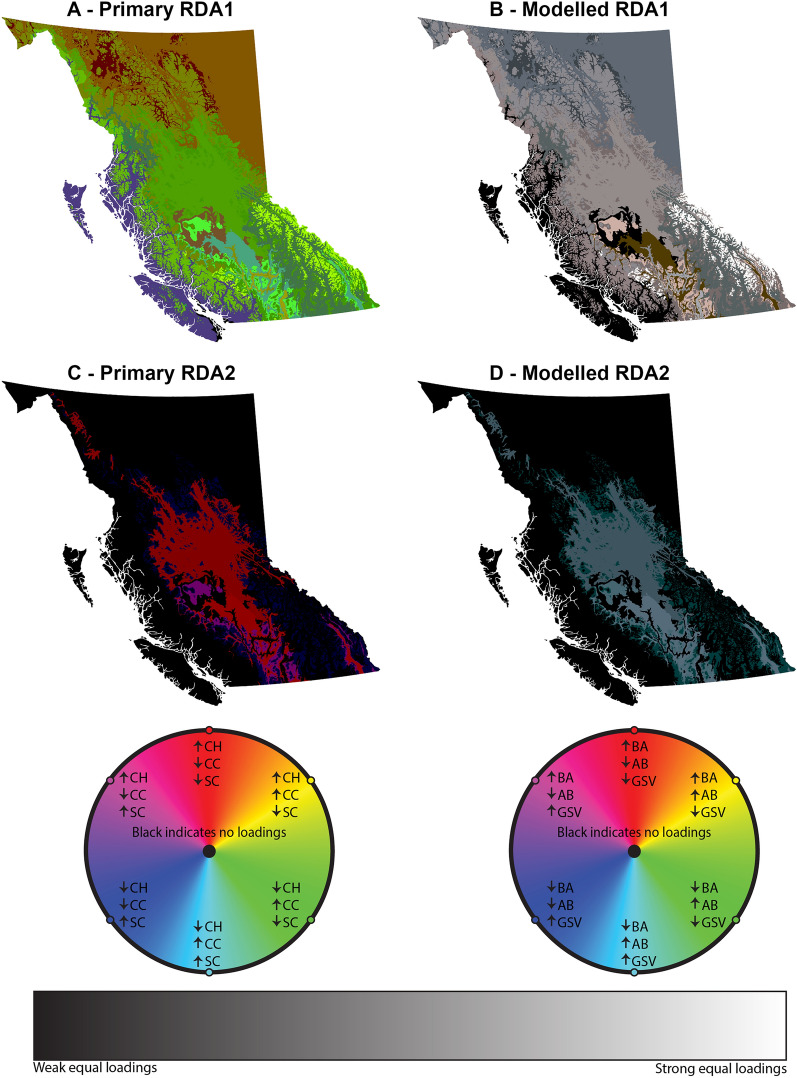
False colour maps by biogeoclimatic ecosystem classification (BEC) zone of axis loadings for the first redundancy analysis (RDA) axis (**A,B**) and second RDA axis (**C,D**). Colour values are normalized to the maximum loading of each variable. (**A,C**) Show axis loadings for canopy height (CH; red), canopy cover (CC; green) and structural complexity (SC; blue). (**B,D**) Show axis loadings for basal area (BA; red), total aboveground biomass (AB; green) and gross stem volume (GSV; blue). Black indicates no significant loadings, while greyscale colours indicate similar loadings across the three variables. Annotated colour wheels for each set of variables also shown. Maps were generated in R version 4.3.1 using the ggplot2 version 2.1.0^99^ and sf version 1.0-14^100^ packages.

Stratification by forest type and ecosystem indicated varying loadings between the RDA axes and predictor/response variables (Fig. 5). All DHI variables had high loadings on RDA1 (similar to the overall dataset), with positive cumulative and minimum DHI loadings, and negative variation DHI loadings. Colder BEC groups (Montane, North, and Alpine) had smaller minimum DHI loadings, which were half as large as the other DHI loadings. As such, we identify the first axis as being consistently associated with overall productivity. The predictor loadings for canopy cover and the modelled forest structural attributes were generally similar within the same stratification/BEC groups. The mixed wood forest and Coastal BEC group had no significant loadings from modelled forest structural attributes for RDA1. Examining the predictor loadings for RDA1 spatially across the province shows a geographic pattern in the loading strengths of primary forest structural attributes (Fig. 6A). The interior (Northern and Southern BEC groups) of the province generally showed high canopy cover loadings, while the Boreal zones (Boreal White and Black Spruce and Sub-Boreal Pine—Spruce) in the northwest had large canopy height loadings. Coastal zones were driven similarly by canopy height and structural complexity, with very low or non-significant loadings from canopy cover (Figs. 5, 6A).

Only four of sixteen BEC zones had a second RDA axis, with none being found in Coastal or Alpine BEC zones (Figs. 5, 6). Two Montane BEC zones had a secondary axis, while the Northern and Southern interior groups each had one zone with a secondary axis. RDA2 often showed the largest absolute loadings in the minimum DHI, which was frequently, but not always, negative (Fig. 5; see Supplementary Information). The Montane group had the largest absolute minimum and variation DHI loadings, which were notably the opposite sign of all other groups with a secondary axis. RDA2 had lower absolute predictor loadings (maximum of ~ 0.25) when compared to RDA1 (maximum of ~ 0.5), with infrequently significant loadings for canopy cover and structural complexity (Fig. 5). There was no consistent loading pattern found in RDA2 for the primary predictor variables (Figs. 5, 6C). Similar to RDA1, the modelled forest structural attribute loadings were generally similar to one another (Figs. 5, 6D).

## Discussion

Relationships between forest ecosystem structure and function are being examined more frequently due to advances in data collection methods, such as remote sensing^26,83,84^. This paper assesses the complementarity of two potential ecosystem-scale EBV indicators for ecosystem monitoring across BC, Canada. Redundancy analysis was used to determine the information overlap of wall-to-wall maps of forest structural attributes and the DHIs, which were both generated from Landsat imagery. This analysis revealed a weak relationship between the two sets of remote sensing derived indicators, with only 15.7% of the variation in the DHIs being explained by the forest structural attributes (Fig. 3). This relationship was also assessed across the different forest classes and ecosystems of British Columbia, and similar results were found (Fig. 4). The small amount of overlap between the two datasets suggests that they are suitable for use in ecosystem monitoring programs, despite both being derived from Landsat imagery.

We also evaluated whether primary forest structural attributes provide more information than modelled forest structural attributes for selecting indicators within a single EBV class. It was found that standard lidar-derived measurements, such as canopy height, canopy cover, and structural complexity (as proposed by Valbuena et al.^9^, contribute the majority of the explained variation in the DHIs, either independently or in their overlap with the modelled forest structural attributes. This could be expected as modelled forest structural attributes are generally directly based on the primary forest structural attributes^12^, however, the creation of modelled forest structural attributes involves modelling expertise and processing time overhead^21,22,85^. One advantage of interpreted products is their potential uptake by a broader set of users and their ability to be closely linked with management actions and monitoring results. This is exemplified by the use of forest biomass monitoring for climate change mitigation^85^. While either set of forest structural attributes may be relevant for a given monitoring task, it is important to note that the primary structural attributes contain the majority of the variation across both classes of structural attributes (Fig. 4). Additionally, the modelled structural attributes are generally not related to structural complexity, which has often been found to be strongly related to biodiversity metrics^86^. Therefore, we generally recommend using the primary forest structural attributes.

Our findings indicate that remote sensing-derived forest ecosystem structure and forest ecosystem function EBVs can be applied in a complementary fashion for ecosystem monitoring^87^, despite both EBVs using surface reflectance values from the Landsat series of satellites in either their calculation or imputation^21,22,26^. While we assessed the complementarity of ecosystem structure and function, a single pair of EBVs measurable at the ecosystem scale, other EBV class combinations at finer scales should also be assessed for complementarity. This is especially relevant when remote sensing datasets such as Landsat can generate a large number of EBVs in different classes^5^. In addition, Skidmore et al.^5^ highlighted that many satellite-derived biodiversity products can inform upon multiple EBV classes simultaneously—e.g., leaf area index being recognized as a ecosystem structure, ecosystem function, and species trait EBV indicator—depending on the spatial scale of analysis and goal of the monitoring program. As such, it is relevant to consider which EBV classes biodiversity products can inform, for given spatial and temporal extents. Other EBVs, such as those found in the community composition and species population classes may also be informed by these products. Further, their reliance on species level data also means that they could be inherently correlated with one another.

In the axis loadings we found a strong linkage between canopy cover, structural complexity, and the DHIs, which was expected as these metrics are more directly related to photosynthesis than canopy height^36^. We also found that axis loadings for modelled forest structural attributes were more similar to canopy cover and structural complexity than canopy height (Fig. 3). This was unexpected as while the modelled forest structural attributes are generally correlated due to their creation methods, these modelled attributes are primarily generated using canopy height information^85^, with relationships often based on tree allometry rather than leaf volume or placement^88,89^.

Forest types with deciduous trees (namely broadleaf and mixed wood) had large, negative structural complexity loadings when compared to the other forest structural attributes (Fig. 5), with forest types having no modelled structural attributes associated with the DHIs. This structural complexity-DHIs linkage may be due to understory cover providing additional photosynthesis in these forest types^90^. In coniferous forests, on the other hand, the DHIs show a weaker relationship with structural complexity than all other forest structural attributes. Notably, the Alpine, Montane, and Northern BEC groups show decoupling of the three DHIs (Fig. 5), with lower loadings between the Minimum DHI and RDA1. This decoupling in the DHIs is potentially due to the near certainty of a pixel containing snow cover over the course of a year leading to consistently low minimum DHI values across the ecosystem, and thus to weaker minimum DHI loadings^47,91^. These ecosystems with DHI decoupling also showed relatively strong relationships to canopy cover and weak relationships with structural complexity. This weak relationship to structural complexity may be due to the dominance of coniferous forests in these ecosystems, which have been shown to have lower forest structural complexity across the globe^86^, with weaker relationships between canopy height and structural complexity being found than deciduous forests^92^.

BC shows varying forest structural drivers of the DHIs depending on the geographic location (Fig. 6). The interior of BC is drier and warmer, and also has productivity being strongly driven by canopy cover, while the Coastal BEC zone group has very low canopy cover loadings and high structural complexity loadings (Fig. 5). The climate of these regions could be influencing the DHIs^93^, leading to varying loadings strengths depending on the distance to the coast. For example, water availability has been shown to be a driver of canopy height^94^. In these wetter regions such as the Coastal group, structural variability is more likely to be present in the vertical, rather than horizontal direction, especially given the relationship between canopy height and structural complexity^92^. Further, the weakest relationships between structure and function were found in the Coastal group of BEC zones (Fig. 4), which may be due to the consistently high amounts of canopy cover and lower amounts of snow found in these zones, leading to potentially saturated NDVI values throughout the year. These consistent forest structural metrics—such as the high canopy cover values found in the Coastal BEC zones—may also lead to lower amounts of variation explained by these metrics, leading to them having smaller loadings, and other metrics with higher variation in their values, such as structural complexity, having larger loadings (Fig. 5). These complex linkages between climate, forest structure, and forest productivity become further complicated by the extreme environmental and topographic gradients within BC’s ecosystems^60,63^, which may warrant study into the scale of these interactions, with a finer (or non-climate based) ecoregionalization potentially revealing different relationships between ecosystem structure and function. Further, when examining these relationships through remote-sensing derived indicators, we recognize that they may change over time and space in response to disturbances, both anthropogenic and natural. For example, modifying the biomass of an area through forest clearing, fires, or insect disturbances may disrupt the underlying assumptions of the DHIs, as abrupt changes in the middle of the year may lead to low Minimum DHI values, high Variation DHI values, and lower than normal Cumulative DHI values.

Various relationship directions and strengths have been found between forest structure and forest function^83^. Temperate and boreal forests often have mixed or negative structure–function relationships, while tropical forests often have positive relationships^83^. We found that indicators of forest structure and function were not strongly linked to one another, both across the full study area and when stratified by ecosystem and forest type (Fig. 5). The forest type models showed higher amounts of variation explained, which may indicate the linkages between forest structure and function are stronger at finer spatial scales, potentially driven by finer scale landscape patterns such as terrain and land cover, rather than climate^95^. Notably, the BEC zones and forest types are delineated at different spatial scales; forest types are mapped at a 30 m pixel size^62,75^, and can be found across the entire province, while individual BEC zones do not span the entirety of the province, and are mapped at a much coarser scale^60^.

Across British Columbia, the DHIs have been shown to be strongly correlated with one another (Fig. 3). While this does imply that using a single one of the three DHI axis may be suitable within this geographic region, the DHIs do provide unique information to one another, which can be especially relevant when stratifying the study area. High levels of minimum DHI, for example, may indicate winter forage availability, which is difficult to capture in the variation or cumulative DHIs^47^. The variation DHI has been shown to be strongly related to seasonality, a key predictor of avian species richness, especially in migratory species^96^. In Coastal ecosystems, which are characterized by mild winters and high precipitation, we found that forest structure and the DHIs are strongly decoupled, with very little variance explained in the DHIs by the forest structural attributes (Fig. 4). This indicates that the complementarity of the DHIs and forest structural attributes should remain, even when expanding the analysis outside of ecosystems with little to no snow cover. Future research should continue to examine the complementarity of various EBV pairs, across varying ecosystems and geographic locations.

A potential limitation of our study is due to the uncertainty inherent to remotely sensed data. Wall-to-wall forest structural information was produced by imputing lidar-derived metrics over satellite spectral reflectance and geospatial data across the forested landscapes of Canada. The accuracy of the forest structure dataset varied between forest structural metrics^21,22^. Likewise, spectral vegetation indices, as discussed, are not direct measurements of productivity, but rather are based on surface reflectance of vegetation, which can be influenced by many factors such as soil, canopy closure, viewing geometry, and atmospheric effects^33,36^. These uncertainties may weaken the relationship between these remote sensing indicators of forest structure and function. Further, while forest structure and function have both been shown to be drivers of biodiversity^8,26^, there are ecological processes that cannot be assessed through remotely sensed data^97^, including predation and fecundity, and thus in situ data are needed to monitor these processes. Finally, our study assumes that structure and function are indicators of biodiversity in this system, and we draw on published demonstrations of the relevance of each of them to species richness and composition, but we recommend further evaluations of these indicators with biodiversity observations to determine the relative contributions of each to estimating species richness and turnover^98^.

## Conclusion

We used redundancy analysis and variation partitioning to assess the complementarity of two potential EBV datasets—forest structure and the DHIs. We also separated the forest structure indicator datasets into primary and modelled structure metrics in order to assess the need to develop more complex structure variables, or if the primary forest structural attributes are suitable on their own. We found that the structure metrics were not strongly related to the DHIs, indicating that they are suitable to be used together as complementary ecosystem-scale EBVs when monitoring forest environments. It was also found that variation in the DHIs explained by the overlap between primary and modelled structure variables was often higher than the variation explained by either individually. The exception to this was found in non-coniferous forest types, Coastal BEC zones, and the BC-wide analysis. These ecosystem structure and function indicators are often attainable at global scales using satellite remote sensing, and recent advances are allowing them both to be generated at medium spatial resolutions. We suggest that biodiversity researchers focus on using forest structural attributes derived directly from the lidar data, and if needed, use a single modelled forest structural attribute, such as total aboveground biomass. In addition, we highlight that the usage of intra-annual summarizations of productivity provide novel information for biodiversity monitoring when used with forest structural attributes as complementary EBVs for a holistic monitoring system for forest ecological integrity.

### Supplementary Information

Supplementary Information.

## Data Availability

The 30 m spatial coverages of the NTEMS data are available online at https://opendata.nfis.org/mapserver/nfis-change_eng.html. BEC zone boundaries were obtained using the *bcmaps* R package. The Dynamic Habitat Indices were calculated using freely available Landsat imagery in Google Earth Engine. Details on their production can be found in Razenkova et al.^45^. All code used for data manipulation and analysis can be found at https://github.com/emuise/code-structProdSem.
