# Supplementary material for: Disentangling linkages between satellite-derived indicators of forest structure and productivity for ecosystem monitoring
Source: Sci Rep. 2024 Jun 14;14:13717. doi: 10.1038/s41598-024-64615-2 (PMC11178816; doi:10.1038/s41598-024-64615-2)
Supplement: Supplementary file 1 — Supplementary Information. [file 41598_2024_64615_MOESM1_ESM.docx]

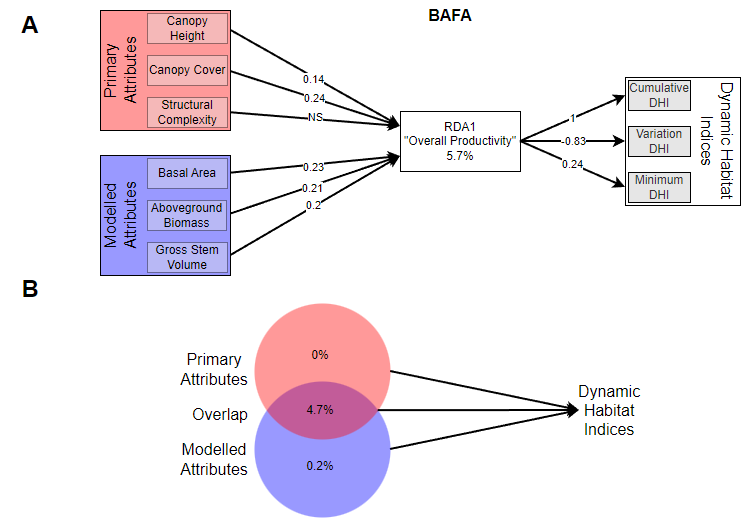


Figure S1 A) Axis loadings from redundancy analysis (RDA) of primary and modelled forest structure variables on the dynamic habitat indices (DHIs). B) Results from variation partitioning of primary and modelled forest structure variables on the DHIs. Both visualized analyses are for the Boreal Altai Fescue Alpine biogeoclimatic ecosystem classification zone.


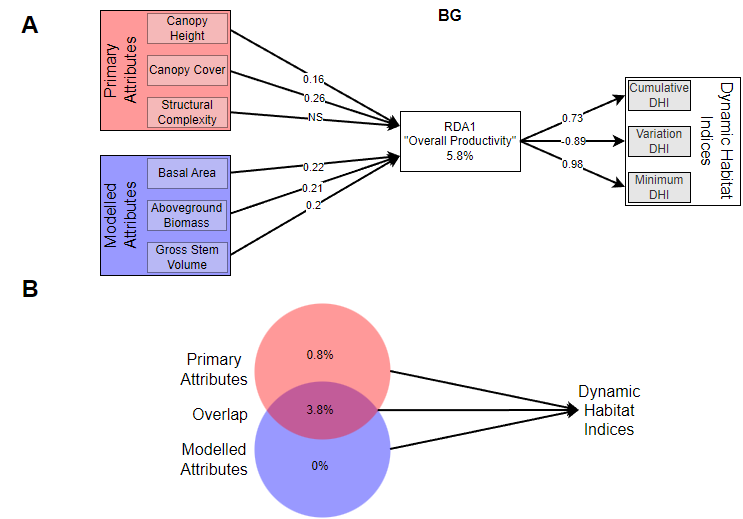


Figure S2 A) Axis loadings from redundancy analysis (RDA) of primary and modelled forest structure variables on the dynamic habitat indices (DHIs). B) Results from variation partitioning of primary and modelled forest structure variables on the DHIs. Both visualized analyses are for the Bunchgrass biogeoclimatic ecosystem classification zone.


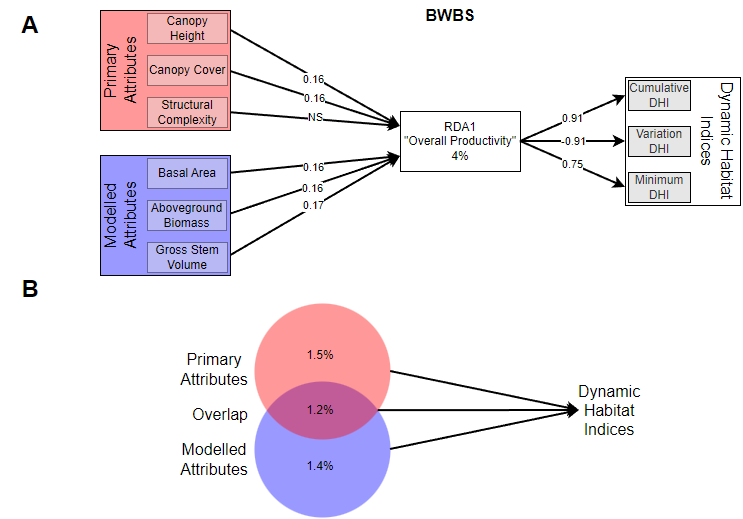


Figure S3 A) Axis loadings from redundancy analysis (RDA) of primary and modelled forest structure variables on the dynamic habitat indices (DHIs). B) Results from variation partitioning of primary and modelled forest structure variables on the DHIs. Both visualized analyses are for the Boreal White and Black Spruce biogeoclimatic ecosystem classification zone.


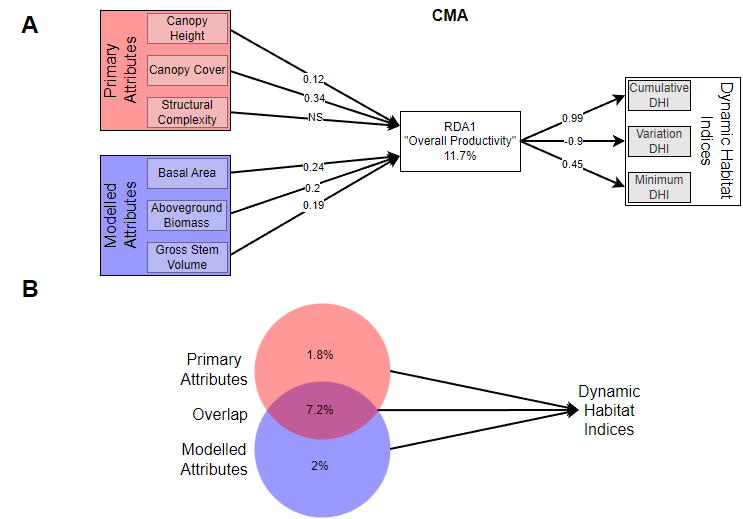
 Figure S4 A) Axis loadings from redundancy analysis (RDA) of primary and modelled forest structure variables on the dynamic habitat indices (DHIs). B) Results from variation partitioning of primary and modelled forest structure variables on the DHIs. Both visualized analyses are for the Coastal Mountain-heather Alpine biogeoclimatic ecosystem classification zone.


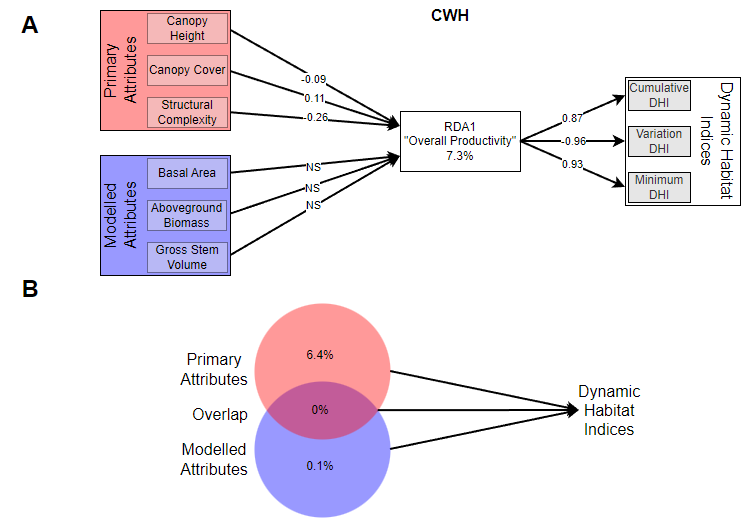


Figure S5 A) Axis loadings from redundancy analysis (RDA) of primary and modelled forest structure variables on the dynamic habitat indices (DHIs). B) Results from variation partitioning of primary and modelled forest structure variables on the DHIs. Both visualized analyses are for the Coastal Western Hemlock biogeoclimatic ecosystem classification zone.


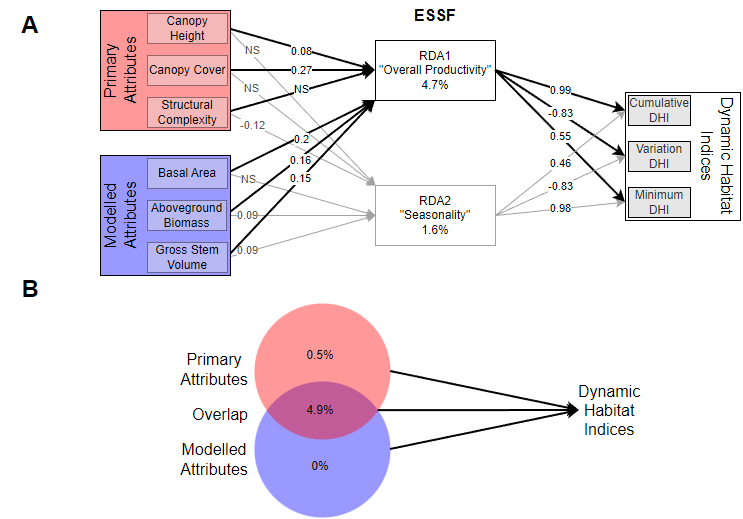


Figure S6 A) Axis loadings from redundancy analysis (RDA) of primary and modelled forest structure variables on the dynamic habitat indices (DHIs). B) Results from variation partitioning of primary and modelled forest structure variables on the DHIs. Both visualized analyses are for the Engelmann Spruce -- Subalpine Fir biogeoclimatic ecosystem classification zone.


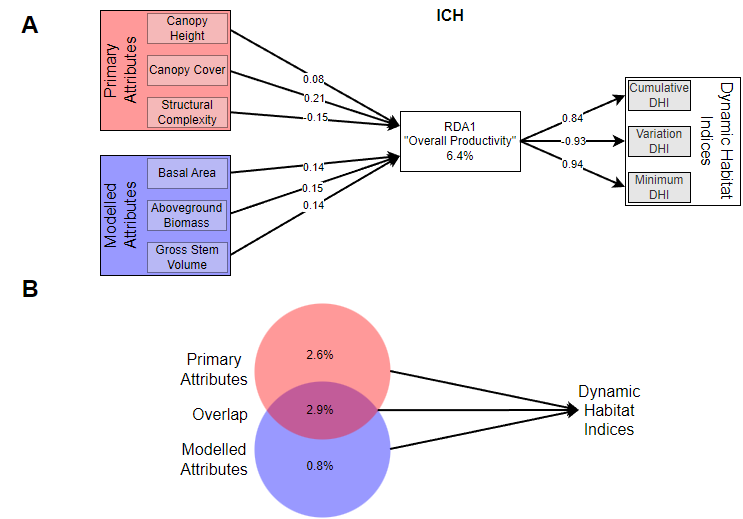
 Figure S7 A) Axis loadings from redundancy analysis (RDA) of primary and modelled forest structure variables on the dynamic habitat indices (DHIs). B) Results from variation partitioning of primary and modelled forest structure variables on the DHIs. Both visualized analyses are for the Interior Cedar -- Hemlock biogeoclimatic ecosystem classification zone.


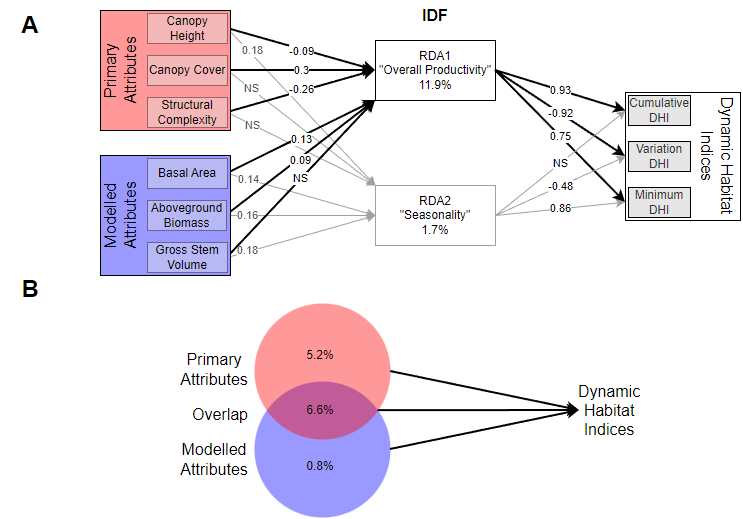


Figure S8 A) Axis loadings from redundancy analysis (RDA) of primary and modelled forest structure variables on the dynamic habitat indices (DHIs). B) Results from variation partitioning of primary and modelled forest structure variables on the DHIs. Both visualized analyses are for the Interior Douglas-fir biogeoclimatic ecosystem classification zone.


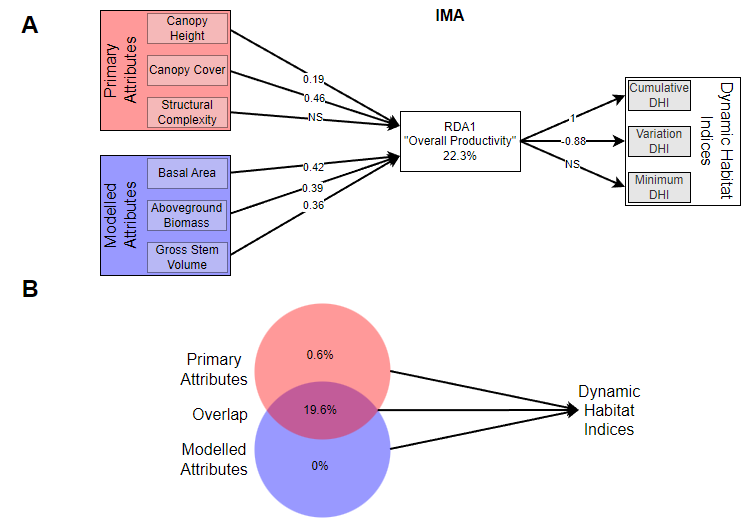


Figure S9 A) Axis loadings from redundancy analysis (RDA) of primary and modelled forest structure variables on the dynamic habitat indices (DHIs). B) Results from variation partitioning of primary and modelled forest structure variables on the DHIs. Both visualized analyses are for the Interior Mountain-heather Alpine biogeoclimatic ecosystem classification zone.


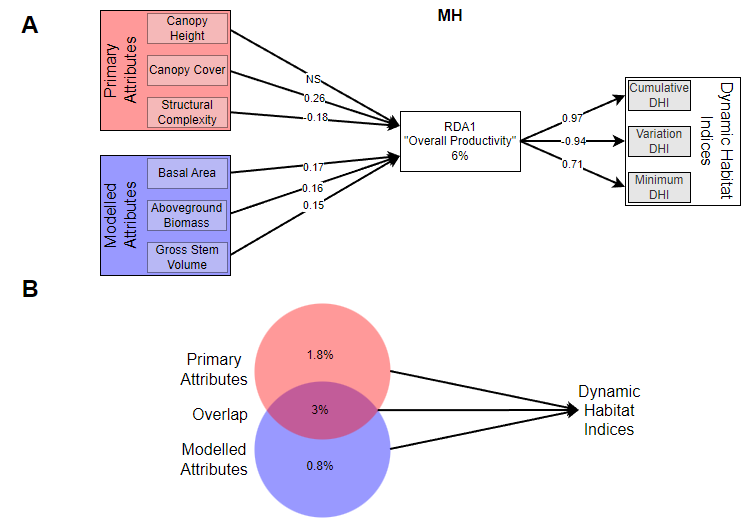


Figure S10 A) Axis loadings from redundancy analysis (RDA) of primary and modelled forest structure variables on the dynamic habitat indices (DHIs). B) Results from variation partitioning of primary and modelled forest structure variables on the DHIs. Both visualized analyses are for the Mountain Hemlock biogeoclimatic ecosystem classification zone.


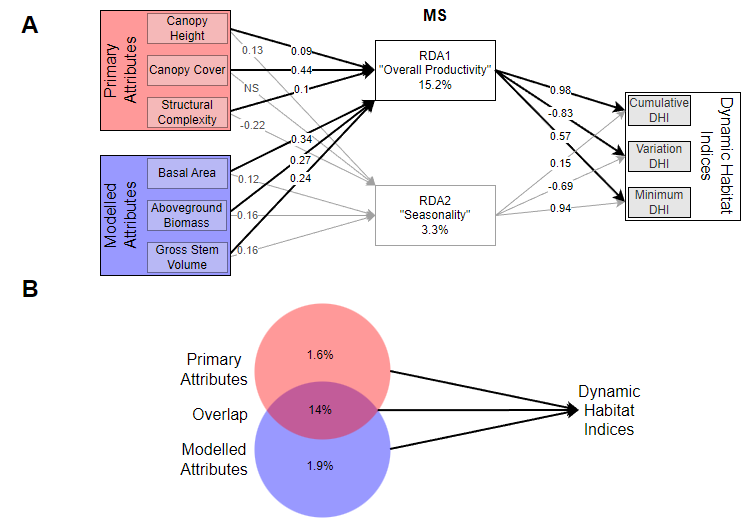


Figure S11 A) Axis loadings from redundancy analysis (RDA) of primary and modelled forest structure variables on the dynamic habitat indices (DHIs). B) Results from variation partitioning of primary and modelled forest structure variables on the DHIs. Both visualized analyses are for the Montane Spruce biogeoclimatic ecosystem classification zone.


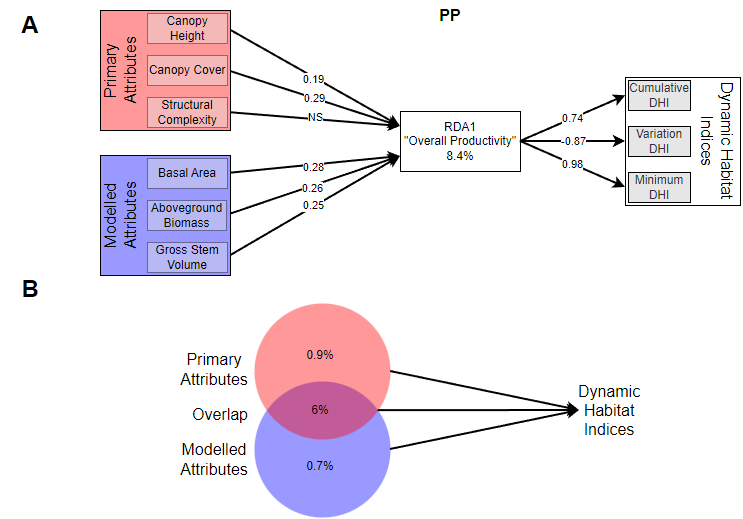


Figure S12 A) Axis loadings from redundancy analysis (RDA) of primary and modelled forest structure variables on the dynamic habitat indices (DHIs). B) Results from variation partitioning of primary and modelled forest structure variables on the DHIs. Both visualized analyses are for the Ponderosa Pine biogeoclimatic ecosystem classification zone.


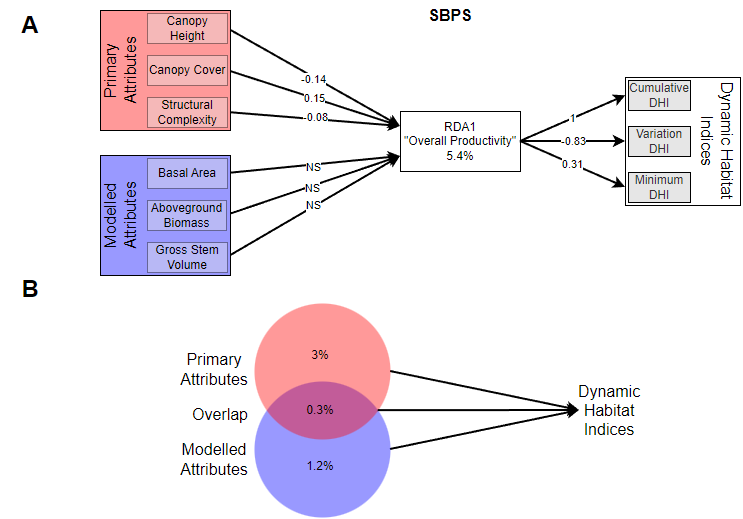


Figure S13 A) Axis loadings from redundancy analysis (RDA) of primary and modelled forest structure variables on the dynamic habitat indices (DHIs). B) Results from variation partitioning of primary and modelled forest structure variables on the DHIs. Both visualized analyses are for the Sub-Boreal Pine -- Spruce biogeoclimatic ecosystem classification zone.


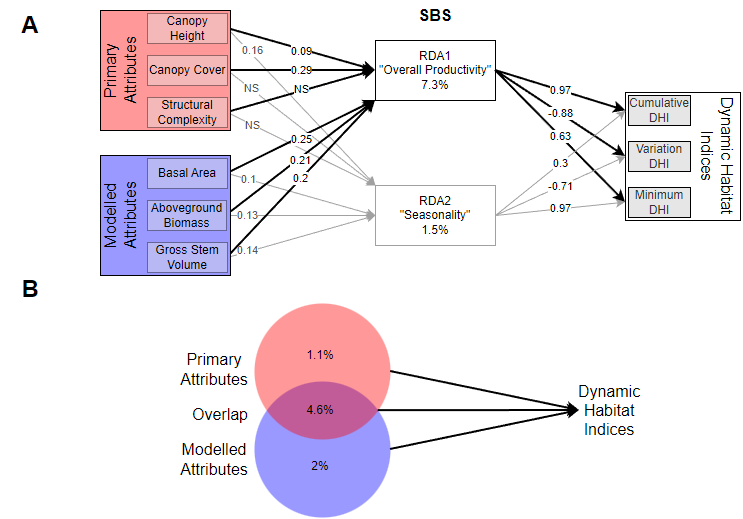


Figure S14 A) Axis loadings from redundancy analysis (RDA) of primary and modelled forest structure variables on the dynamic habitat indices (DHIs). B) Results from variation partitioning of primary and modelled forest structure variables on the DHIs. Both visualized analyses are for the Sub-Boreal Spruce biogeoclimatic ecosystem classification zone.


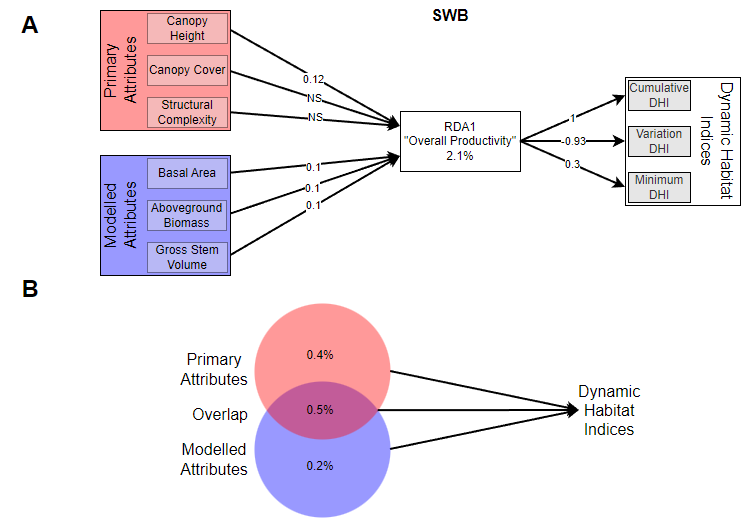


Figure S15 A) Axis loadings from redundancy analysis (RDA) of primary and modelled forest structure variables on the dynamic habitat indices (DHIs). B) Results from variation partitioning of primary and modelled forest structure variables on the DHIs. Both visualized analyses are for the Spruce -- Willow -- Birch biogeoclimatic ecosystem classification zone.


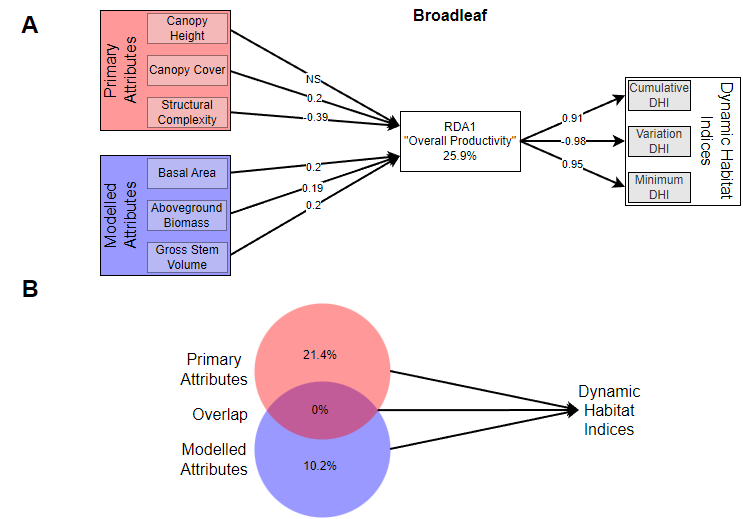


Figure S16 A) Axis loadings from redundancy analysis (RDA) of primary and modelled forest structure variables on the dynamic habitat indices (DHIs). B) Results from variation partitioning of primary and modelled forest structure variables on the DHIs. Both visualized analyses are for the Broadleaf forest type.


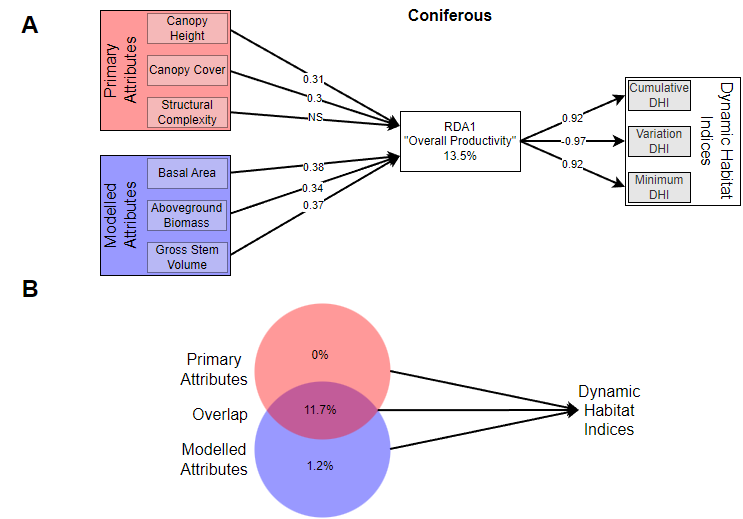


Figure S17 A) Axis loadings from redundancy analysis (RDA) of primary and modelled forest structure variables on the dynamic habitat indices (DHIs). B) Results from variation partitioning of primary and modelled forest structure variables on the DHIs. Both visualized analyses are for the Coniferous forest type.


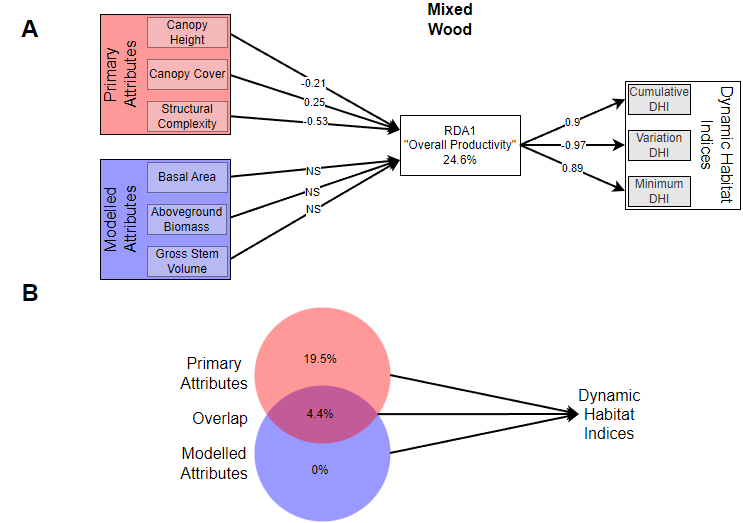


Figure S18 A) Axis loadings from redundancy analysis (RDA) of primary and modelled forest structure variables on the dynamic habitat indices (DHIs). B) Results from variation partitioning of primary and modelled forest structure variables on the DHIs. Both visualized analyses are for the Mixed Wood forest type.


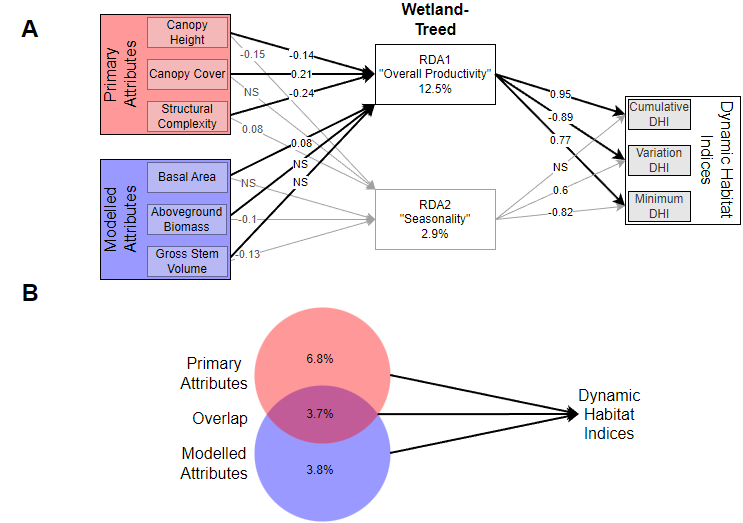


Figure S19 A) Axis loadings from redundancy analysis (RDA) of primary and modelled forest structure variables on the dynamic habitat indices (DHIs). B) Results from variation partitioning of primary and modelled forest structure variables on the DHIs. Both visualized analyses are for the Wetland-Treed forest type.

**
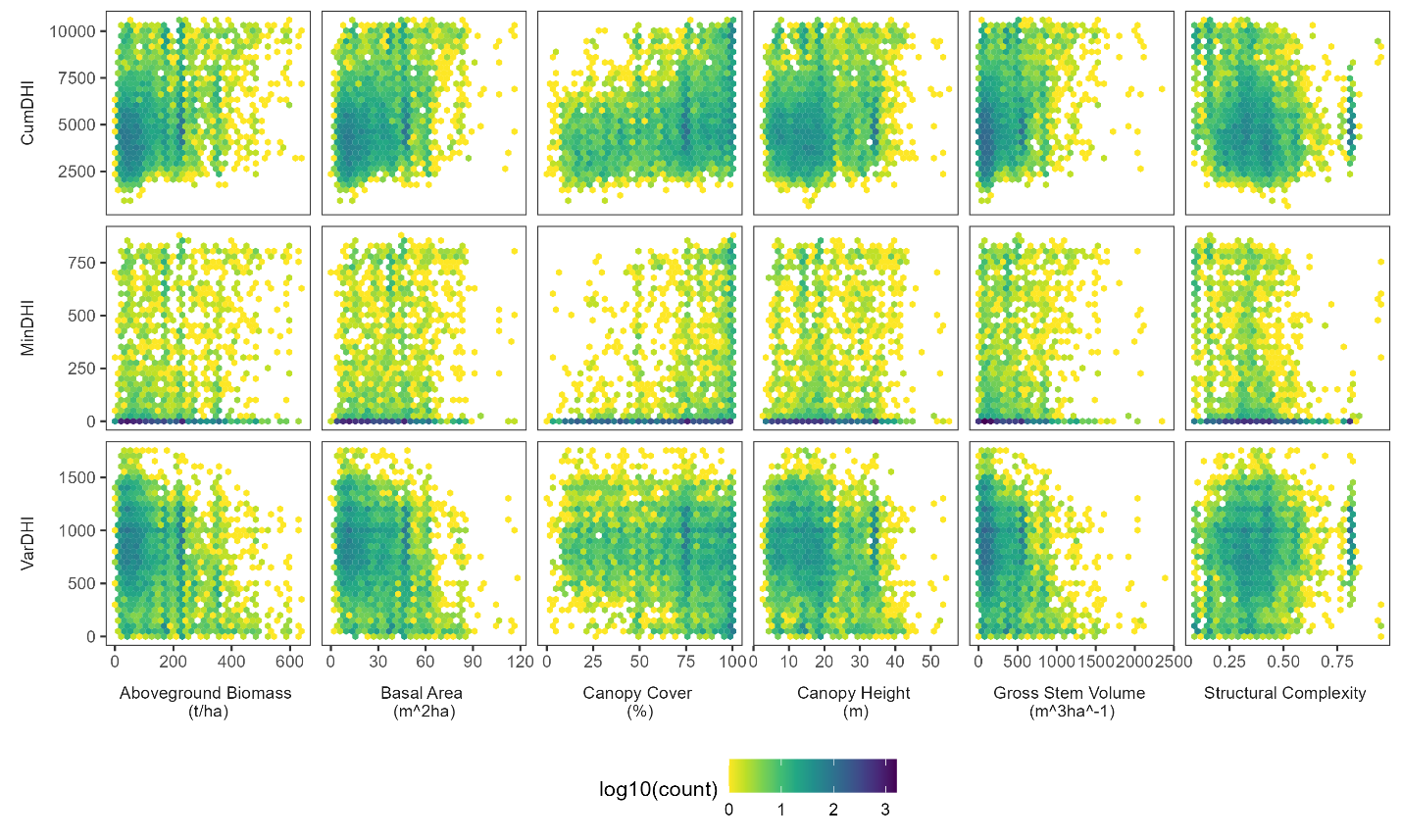
**

Figure S20 Density hexplots of forest structural attributes and the Dynamic Habitat Indices. Note the log scale.
